# Antisemitism in Medicine: An International Perspective

**DOI:** 10.5041/RMMJ.10536

**Published:** 2025-01-30

**Authors:** Michael Gordon, Jerome Teitel, Ted Rosenberg, Ruth Oratz, Naomi Katz, David Katz

**Affiliations:** 1 Department of Medicine, University of Toronto, Toronto, Canada; 2 Department of Family Medicine, University of British Columbia, Victoria, Canada; 3 Department of Medicine, New York University Grossman School of Medicine, New York, USA; 4 Department of Pediatrics, University of Melbourne, VIC Australia, Melbourne, Australia; 5 Department of Medicine, Division of Immunoloy, University College London, London, United Kingdom

**Keywords:** Antisemitism, healthcare impact, resurgence

## Abstract

Throughout history, Jewish people have long been recognized for their achievements in the world of medical science. For example, prior to the Holocaust, many outstanding physicians in Germany were Jewish. However, even in the 1930s, refugee European Jewish doctors faced significant barriers when they tried to escape and practice elsewhere because of long-standing prejudices and anti-Jewish quotas in medical schools and hospitals around the world. Eventually quotas fell, and the period after World War II once again saw a tremendous growth in numbers of Jews excelling in medicine internationally. Since the Hamas attack on Israel on October 7, 2023, there has been a resurgence of antisemitism worldwide. It is especially noticeable in the world of healthcare. This article evaluates and highlights examples of antisemitism in four countries by authors from each of these jurisdictions.

## INTRODUCTION

The October 7, 2023, invasion of Israel from Gaza appears to have reawakened a latent antisemitism that appears to exist in many countries. Expressions of antisemitism are either blatant or couched in the more “acceptable” clothes of anti-Zionism. To offer a sense of the *zeitgeist* (the prevailing mood and spirit) surrounding these issues, the International Federation of Medical Student Associations (IFMSA) recently suspended the Federation of Israeli Medical Students from its organization in an arbitrary process. Founded in 1951, the IFMSA has national member organizations in 137 countries including China, Russia, and Syria. These latter countries have notably poor human rights records yet have remained members in good standing without undergoing similar scrutiny or sanctions as applied to Israel.

Hence, within this difficult context, we explore some recent experiences and reflections of Jewish physicians from four countries: Canada, the United Kingdom (UK), Australia, and the United States (US). Sadly, their experiences are mostly congruent and illustrate the existence and degree of antisemitism and anti-Zionism expressed toward healthcare providers in their respective countries.

Given the sense of the mission of medicine expressed by medical professionals, one might have thought that healthcare, given its lofty and oft-stated aspiration to care for the individual, regardless of sex, race, color, or creed, might be immune to the virus of racism.

As pointed out in a recent article, “A wave of open Jew-hatred by medical professionals, medical schools, and professional associations in the wake of the Hamas slaughter suggests that a field entrusted with healing is becoming a licensed purveyor of hatred.”^1^ Sadly, when it comes to Jews and antisemitism, a hope that healthcare might be immune to this social virus has not been realized.

Each author provided anecdotes from their home country (Canada, UK, US, and Australia) to document evidence of antisemitism and anti-Zionism which, while not new historically, has become more virulent since the outbreak of the October 7, 2023 conflict. To that end, the authors refer to up-to-date articles in the professional and lay media and the publicly available comments of politicians and members of professional organizations. Where available, opinion polls are cited. We were able to document evidence of systematic antisemitic actions and personal attacks on Jewish medical students and physicians in all four countries.

Each of the authors has witnessed anti-Jewish prejudice throughout all levels of the healthcare system (from medical students, residents, teachers, members of administration), and in workplaces and medical associations. However, for this paper the examples offered primarily relate to one of these aspects in each country.

## ANECDOTAL ACCOUNTS OF THE AUTHORS

### Canada—Faculty Members

Antisemitism has a long history in Canada. According to the noted Canadian historian Irving Abella: “Until the 1950s it [antisemitism] had respectability; few people apologized for being anti-Jewish. Expressions of antisemitism were heard in the halls of Parliament, read in the [daily] press, taught in some schools and absorbed in some churches.”^2^ He goes on to explain that, during the 1920s and 1930s, Canadian Jews were quite used to quotas and restrictions. Jewish people experienced hiring discrimination both in industry and academia, and “Jewish doctors could not get hospital appointments.”^2^

Mount Sinai Hospital in Toronto was established because no other hospital in Toronto would hire Jewish physicians.^3^,^4^ In 2022, the College of Physicians and Surgeons of Ontario (Canada’s most populous province) published an article on the historical roots of the ongoing problem of antisemitism in Canada.^5^ They discuss an incident that occurred in Montreal:

In the 1930s, doctors in hospitals across Montreal walked off the job to protest the hiring of a Jewish senior intern, Dr. Samuel Rabinovitch. The strike ended four days later when Dr. Rabinovitch resigned his position because he was worried about the effect on patient care. It was not until 1951 that the first Jewish doctor—Dr. Barnet Berris—was granted a full-time faculty position within the University of Toronto’s Department of Medicine.^5^

Relating to faculty members, Dr Ayelet Kuper, Senior Advisor on Antisemitism at the Faculty of Medicine of the University of Toronto (U of T), recently described a disturbing complaint submitted by several of the U of T faculty members. She wrote: “[the] allusion to the long-standing myth of ‘Jewish power’ was only one of many antisemitic aspects of the leaked complaint letter … [even today] Jews are routinely accused of controlling the media, the economy, and the actions of major nation-states.”^6^^(p162)^

In British Columbia, Canada’s westernmost province, social media posts vilifying Israel and espousing Jew hatred were circulated by physicians at the Faculty of Medicine of the University of British Columbia (UBC), after the October 7 massacre. Allegations included Christ-killing, organ trafficking, and other nefarious conspiracies supposedly hatched by Jewish doctors. Some asserted that Jewish faculty should not be allowed to adjudicate resident matching because the examining doctors were Jewish and might be racist. In November of 2023, one-third of all UBC medical students signed a petition endorsing this call. Jewish learners who refused to sign were harassed by staff and students on social media.

When challenged, the Dean of the medical faculty refused to recognize antisemitism as a problem at UBC or to meet with the representatives of almost 300 Jewish physicians who had signed a letter expressing concern about the tolerance of Jew hatred, and the danger of a toxic hyper-politicized academic environment. This led to the public resignation of one of the authors (TR), a senior Jewish faculty member.^7^

A recent article published on December 6, 2024, in the *Toronto Star*, recounts major antisemitic attacks on medical staff:

The JMAO [Jewish Medical Association of Ontario] brought Lee-Segal and several doctors to the Ontario legislature this week to flag a “disturbing rise in antisemitism” experienced by some Jewish medical practitioners in the wake of the Hamas attack on Israel on Oct. 7, 2023 and the increasingly deadly war in Gaza.

“Many of our members have been doxxed and subjected to targeted harassment simply for being Jewish,” association president Dr. Lisa Salamon told a news conference Wednesday.

These attacks have profound mental health impacts and send a chilling message to all Jewish professionals in health care.^8^

### Australia—The Workplace

Alarming gaps in cultural safety have emerged in healthcare in Australia, mirroring what Kingsbury and Greene have described. One *Guardian* news article states: “Amid the turmoil, rates of antisemitism targeting Australia’s 100,000 Jews have leapt since the attacks of 7 October.”^9^ The article reported that data gathered by the Executive Council of Australian Jewry (ECAJ) showed that in the six months from October 1, 2023 to March 31, 2024 there was a 427% increase in the number of anti-Jewish incidents compared to the same period the year before; in the immediate aftermath of October 7, incidents shot up by 738%.^9^ As noted in the earlier report, a “wave of open Jew-hatred by medical professionals, medical schools, and professional associations in the wake of the Hamas slaughter suggests that a field entrusted with healing is becoming a licensed purveyor of hatred.”^1^^(p332)^

**Figure 1:**
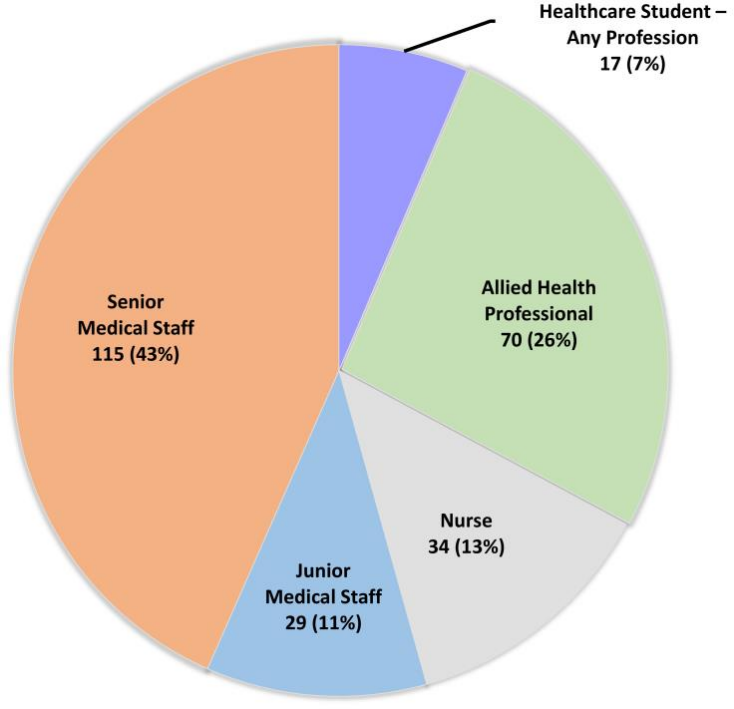
Professional Background of Respondents Surveyed About Experiences of Antisemitism in the Workplace since October 7, 2023. Data taken from the Australian Academic Alliance Against Antisemitism in Healthcare.^11^

To better understand the impact of antisemitism in the healthcare setting post October 7, a cross-sectional electronic survey of Jewish healthcare students and professionals was conducted in Victoria, Australia.^10^ Initial distribution of the link was primarily through synagogue networks and support groups for Jewish health professionals that were established after October 7, 2023. The survey link was open for three months, between December 2023 and February 2024. Data collected were anonymous, with respondents offered the opportunity to provide their details if they wished to be contacted. Of the 265 respondents, most were senior medical staff and allied health professionals (Figure 1); 68% worked in the hospital setting, 65% of whom were in the public health system.^11^

Chillingly, since October 7, 2023, almost half (127) of those in the health field have felt the need to hide their Jewish identity; almost two-thirds (162) are aware of someone else feeling this need.^12^ One-third (88) have experienced antisemitism in the workplace, with just under two-thirds feeling unsafe or uncomfortable considering these escalating experiences. Since October 7, nearly 1 in 3 respondents have felt the need to avoid group situations at work or social situations with work colleagues outside of the workplace.^10^ According to a November 24, 2024 report in Australia’s *Financial Review*, “Jews are dismayed by the surge in hatred since October 7 and the lack of leadership by Prime Minister Anthony Albanese.”^13^ According to Jillian Segal, Australia's Special Envoy to Combat Antisemitism, it’s a “fight for our way of life.”^13^

It is hard to imagine any other minority group in Australia tolerating such a situation; nor should it be tolerated.

### United Kingdom—The Workplace

Like the other countries discussed herein, the UK has a long history of prejudice against Jews in medicine. Jews were long kept out of the profession, but by the interwar period increasing numbers of Jewish students were accepted to study medicine. However, they were still denied hospital appointments. This latter form of prejudice only decreased after the establishment of the National Health Service (NHS) in the late 1940s.^14^

During the previous Gaza conflict (2021), four doctors were reported to the General Medical Council (GMC; the UK medical regulator) for antisemitic statements. In 2022, consistent with past data, the NHS staff survey showed that 30% of Jewish staff had experienced discrimination. Early in 2023, the Jewish Medical Association (JMA) reported Dr Martin Whyte, a Newcastle upon Tyne pediatric trainee and British Medical Association (BMA) officeholder (!) to the GMC for antisemitism. Dr Whyte had used social media to propagate the harmful falsehood that the Holocaust was a hoax and that more Jews should have been gassed. To its credit, the BMA has suspended him; to date (November 2024), the GMC “is investigating” and “taking it forward” but has not taken any action.^15^

There has been a dramatic increase of reports of antisemitism to the GMC since October 7, 2023—overall ~15-fold.^16^ Reports made by individuals and other organizations indicate that around 8% of these cases are being investigated, which is consistent with the “GMC standard” level. Of the 28 cases triaged by the Jewish Medical Association (up to December 2024) for breaching both the International Holocaust Remembrance Alliance (IHRA) definition of antisemitism^17^ and relevant GMC good medical practice standards,^18^ 50% are being taken forward; but of this 50%, all except one are still practicing unrestricted while being investigated. Amazingly the unrestricted includes the general practitioner head of the proscribed terrorist organization Hizb ut-Tahrir. As in Australia, a rapid survey of Jewish doctors and other healthcare professionals (November–December 2023) indicated that a majority had experienced antisemitic behavior^19^; 78 separate incidents of antisemitism were reported to the UK Community Security Trust (CST).^16^ A disturbing feature is that UK medical schools and medical students are also targeted in many ways, both person to person (sometimes as part of campus protests) and via social media.^20^ Even induction material for the 2024–5 academic year was not immune: for example, at least one medical school included a link to the Israel boycott guide.^21^

### United States—Campus Wars and Attacks on Healthcare Facilities

Jewish people have lived in America since the 17th century and have faced antisemitism in varying degrees over the years.^22^ Jewish physicians and hospitals often provided healthcare to their own communities to avoid antisemitic discrimination.^23^ As is the case of the other three countries surveyed here, since October 7, 2023, many university campuses and medical schools have become hotbeds of pro-Hamas, pro-terrorist agitation, whilst vilifying Jews and Israel. Social media have been flooded with cries of jubilation from physicians and other healthcare providers that exalt the death and destruction of Jews and Israel.^24^

At many US campuses and healthcare facilities, pro-Hamas demonstrations have sprouted. These are reported to be frightening—both to staff and, even worse, for patients.^24^ In New York, demonstrations have been staged at, among other notable locations, New York University Langone Hospital, Bellevue Hospital, Mount Sinai Hospital, Memorial Sloan Kettering Cancer Center, and the Manhattan Veterans Affairs Medical Center—institutions where many Jewish healthcare workers provide services to many patients, Jewish and non-Jewish alike.^25^,^26^ At the university level, as a frightening example, Columbia has been a locus of intense of antisemitic activity. Similar reports have been received from across the country at numerous campuses.

**Figure 2:**
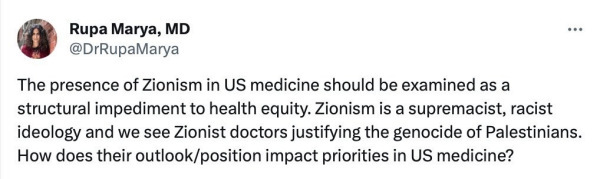
Screen Capture of Message Posted on X by Dr Rupa Marya.^28^

In San Francisco, the University Hospital has seen outright and hateful antisemitism. An article published on June 24, 2024, described the blatant anti-Zionist fervor, reporting that medical students and doctors at one of the nation’s pre-eminent medical schools and teaching hospitals could be heard shouting: “intifada, intifada, long live intifada!”^26^ These chants could be heard by patients in their hospital rooms at the UCSF Medical Center.^27^ The following quote reflects the feelings of those on the receiving end of what can only be defined as old-fashioned Jew hatred: “It’s not the words of my colleague that leave me feeling unwelcome and frankly unsafe here at work,” Dr Avromi Kanal, a hospitalist and assistant professor of medicine explained, “It’s the persistent unwillingness of my leaders to clearly denounce them and ensure my inclusion in this broad community here at UCSF.”^27^ The university did respond to a different post by Dr Rupa Marya, an internist, who in January 2024 wrote the message shown in Figure 2 on X.^28^

In response to all this hostility, Jewish professionals established the American Jewish Medical Association to unite them as a community, to provide information and education, and to stand up against antisemitism in both medicine and academia.^29^

### Similarities Across Countries

Similar expressions of antisemitism have been seen in each country discussed—both at the institutional and the individual level. The background of the authors prevented exploration into related events in non-English-speaking countries. However, despite the different cultures, histories, and healthcare systems (of the four, only the US does not have a universal healthcare system), the experiences and findings in each country were all very similar. There appeared to be reluctance, if not a refusal, to acknowledge that antisemitism and harassment were being experienced by students and staff members identified as Jewish. Furthermore, there was tolerance for what could well be deemed *hate speech*, justified under the concept of freedom of expression.

## DISCUSSION

In Western society in general, and medicine in particular, we believe we are observing a worsening of the ancient scourge of antisemitism. A comprehensive history and detailed explanation of the “Oldest Hatred,” as it has been called by the eminent late Chief Rabbi of Britain, Sir Jonathan Sacks, is clearly beyond the scope of this short account.^30^ However, it is important to point out, as did Rabbi Sacks, that this pathology is not a problem *with* the Jews, but for the Jews and for all humanity. As he put it so pithily, “The hate that begins with Jews never ends with Jews.”^30^ As such, this is not just a question for Jewish patients and healthcare workers, but one for wider society in general and the medical profession in particular. Our observations in each of our home countries over the last year lead us to conclude that the events of October 7, 2023 have served as a pretext to justify or normalize antisemitism that was simmering below the surface. By equating “Jews” with “supporters of Israel” (a reasonable equation given that most people who self-identify as Jews are supportive of the Jewish state), antisemites have fashioned a socially acceptable, ostensibly progressive outlet by which they can express their racist ideology.

A key question remains, as it does in all four surveyed countries: how does the resurgence of antisemitism affect the Jewish community, particularly its students, teachers, and doctors within the society in which they live and work? When and how will the national healthcare authorities devise strategies to combat this unacceptable bigotry? We are forced to ponder how different the responses might be if these levels of animosity had been directed at other groups in society.

History has repeatedly shown that the malignancy of hatred for *any* group does not end with damage to the original target. By the time it has spread, it may well be too late. As such, it behooves us all, especially those of us in the healthcare professions, to take this phenomenon seriously.

### Recommendations for Public Policy

Apart from national efforts meant to combat racism in all its forms, there are many steps the various actors in the health professions might take, the first being documentation of antisemitism and its consequences within medical schools and the health professions. Medical schools and faculties must take steps to organize themselves to gather relevant data. But this will not be enough. It will be a bigger challenge to develop policies that might halt or at least mitigate this damage, and lead to more informed administrators within the governing bodies of medical schools and the medical profession. Our suggestions include:

1. Develop clear definitions of antisemitism that are widely disseminated to all physicians, healthcare providers, staff, and students.
2. Develop clear policies for conduct based on the above policies, and clear consequences if these are violated.
3. Require education regarding antisemitism for leadership, staff, faculty, and students at all medical institutions, schools, universities, and regulatory bodies.

As to the ubiquitous Diversity, Equity, and Inclusiveness (DEI) programs, in recent years much has been done to help protect the interests of other minorities; but antisemitism has generally not been included, as Jews are seen as a privileged group and are therefore characterized as “oppressors” rather than “victims.” These programs must be reformed to include antisemitism within their remit.^31^,^32^ A useful start, among other things, would be inclusion of Jewish members on the relevant committees and working groups. Jews have been excluded from many such groups, which has led to scenarios in which action has not been taken in response to anti-Jewish provocations that would not be tolerated if directed against other identifiable minorities.

Reforms are also needed in medical education. For example, *The Lancet* recently published a report on “medicine, Nazism, and the Holocaust: historical evidence, implications for today, teaching for tomorrow.”^33^ Already in 2019, Richard Horton, the editor-in-chief of *The Lancet*, had offered an anguished cry: “How can it be that in the 21st-century European nations are debating the rise of antisemitism? On the continent torn apart by a fascism that quickly became a genocide, it seems barely credible that antisemitism not only survives but also flourishes in increasingly populist and nationalist democracies. But survive and flourish it does.”^34^^(p105)^ He expands his argument as follows:

Sceptics might argue that Nazi atrocities could never happen again. But as Matthew Wynia and Alan Wells pointed out over a decade ago, an uncomfortable question has to be asked: “How did a professional group that was internationally respected, scientifically innovative, and ethically advanced, evolve an understanding of their ethical, social, and scientific obligations which led them, with only rare exceptions, to use their advanced scientific knowledge and professional ethics to justify committing murder and the most heinous crimes against humanity?” The difficult truth is that it was the profession’s success that contributed to its hubris and collusion with a racist political regime. Medicine today is even more powerful than it was in the 1930s. The risks of abuse are greater now than then. In benign political times, the accrual of such power may be a theoretical danger only. But these are not benign political times. David Grossman notes, in his book *Writing in the Dark* (2008), that the Jewish people share “a profound lack of confidence in the possibility of existence.” In 2019, that confidence might have reached a nadir.^34^^(p105)^

Teaching the Holocaust in medicine would be an act of resistance against depravity and discrimination. Medical Schools and residency programs should include the recommendations of the Lancet Commission in the curriculum of all healthcare trainees, especially but not exclusively physicians.

## CONCLUSIONS

Was the Holocaust a one-off experience or a reflection of latent antisemitism actualized concomitant to events in history? Is antisemitism becoming an acceptable phenomenon in the Western world? These questions were explored via the anecdotal experiences of the authors. Clearly, antisemitism is not going away. It never has. In some ways, it can be likened to an infection that, while deemed to be controlled or cured, finds ways to recur because it mutates enough to evade existing preventions and treatments, and because of human inattention or refusal to understand the history of infection by the public. The analogy to infectious disease is apt. There are still periodic outbreaks of measles, whooping cough, polio, venereal disease, and tuberculosis despite the available tools that can prevent, diagnose, and effectively treat them.^35^ “Anti-vaxxers” in many ways share the same mentality as antisemites, refusing to understand the history and the danger of forgetting the past. As such, antisemitism must be fought resolutely at all levels of society. In our remit, the healthcare sphere, we have tried to indicate through a brief survey of four countries how firmly embedded hatred of and discrimination against Jews exist at all levels: students, teachers, administration, and, perhaps of most concern, patient care. The time is short, and the task is almost overwhelming. But as it is written in *Pirkei Avot* (Ethics of the Fathers) and attributed to Rabbi Tarfon, “It is not your duty to finish the work, but neither are you at liberty to neglect it.”^36^

## Abbreviations

BMA: British Medical Association
DEI: Diversity, Equity, and Inclusion
IFMSA: International Federation of Medical Student Associations
IHRA: International Holocaust Remembrance Alliance
JMA: Jewish Medical Association
NHS: National Health Service
U of T: University of Toronto
UBC: University of British Columbia
UCSF: University of California, San Francisco
UK: United Kingdom
US: United States
